# Biomechanical investigation of a novel ratcheting arthrodesis nail

**DOI:** 10.1186/1749-799X-5-74

**Published:** 2010-10-14

**Authors:** Jeremy J McCormick, Xinning Li, Douglas R Weiss, Kristen L Billiar, John J Wixted

**Affiliations:** 1 Department of Orthopaedic Surgery, University of Massachusetts, Medical Center, Worcester, Massachusetts 01655, USA; 2 Biomedical Engineering Worcester Polytechnical Institute Worcester, Massachusetts 01655. USA; 3 Department of Orthopaedic Surgery, University of Massachusetts, 55 Lake Avenue North, Worcester, Massachusetts 01655, USA

## Abstract

**Background:**

Knee or tibiotalocalcaneal arthrodesis is a salvage procedure, often with unacceptable rates of nonunion. Basic science of fracture healing suggests that compression across a fusion site may decrease nonunion. A novel ratcheting arthrodesis nail designed to improve dynamic compression is mechanically tested in comparison to existing nails.

**Methods:**

A novel ratcheting nail was designed and mechanically tested in comparison to a solid nail and a threaded nail using sawbones models (Pacific Research Laboratories, Inc.). Intramedullary nails (IM) were implanted with a load cell (Futek LTH 500) between fusion surfaces. Constructs were then placed into a servo-hydraulic test frame (Model 858 Mini-bionix, MTS Systems) for application of 3 mm and 6 mm dynamic axial displacement (n = 3/group). Load to failure was also measured.

**Results:**

Mean percent of initial load after 3-mm and 6-mm displacement was 190.4% and 186.0% for the solid nail, 80.7% and 63.0% for the threaded nail, and 286.4% and 829.0% for the ratcheting nail, respectively. Stress-shielding (as percentage of maximum load per test) after 3-mm and 6-mm displacement averaged 34.8% and 28.7% (solid nail), 40.3% and 40.9% (threaded nail), and 18.5% and 11.5% (ratcheting nail), respectively. In the 6-mm trials, statistically significant increase in initial load and decrease in stress-shielding for the ratcheting vs. solid nail (*p *= 0.029, *p *= 0.001) and vs. threaded nail (*p *= 0.012, *p *= 0.002) was observed. Load to failure for the ratcheting nail; 599.0 lbs, threaded nail; 508.8 lbs, and solid nail; 688.1 lbs.

**Conclusion:**

With significantly increase of compressive load while decreasing stress-shielding at 6-mm of dynamic displacement, the ratcheting mechanism in IM nails may clinically improve rates of fusion.

## Background

Intramedullary (IM) implants are used clinically to provide stability and expedite fracture healing and fusion [1-5]. IM devices may be utilized to facilitate femoral-tibial (knee) [3,5-9] or tibio-talo-calcaneal (TTC) fusion [4,10,11]. Knee fusion is most commonly performed for failed total knee arthroplasty secondary to multiple infections or severe post traumatic arthritis [1,5,9,12]. TTC fusion is a salvage procedure performed in patients with severe pain and/or deformity as seen in complex hindfoot fractures or congenital deformities, septic arthritis, failed total ankle arthroplasty, or neuropathic (Charcot) arthropathy [4,11,13]. The goal of fusion surgery is to relieve pain and improve function by eliminating motion through solid bony union at the problem joint [14]. To achieve knee or TTC fusion, techniques such as use of plates, screws, pins, staples, and external fixation devices have all been described in the literature [3,14-18].

Seemingly inherent with the complexity of the procedure is a relatively high rate of complications such as nonunion, delayed union, sepsis, delayed wound healing, and adjacent joint arthritis [2,4,9,13]. Cooper cited an 11-40% rate of nonunion in their TTC fusion study patients [10]. Knee fusions have achieved better success than TTC fusion, however, multiple studies still show a 20-30% failure of fusion depending on the technique that is utilized [7-9,19,20].

**Figure 1:**
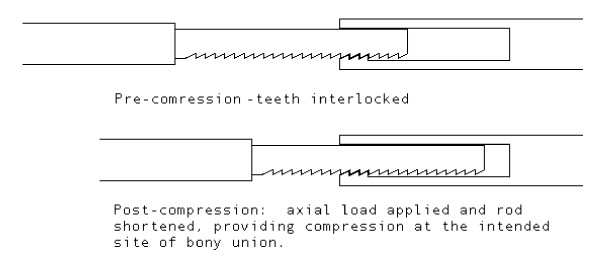
**The ratchet design of the novel arthrodesis nail**. Both pre-compression and post-compression teeth interlocking are demonstrated. Axial loading will result in nail shortening and dynamic compression at the site of fusion.

With these factors in mind, improving mechanical stability at the fusion surface to decrease nonunion rates while minimizing patient morbidity is a difficult endeavor. This novel arthrodesis nail with a ratcheting mechanism (Figure 1) was designed with the goal to maintain maximal compression across the joint fusion surface throughout the healing process, which may theoretically improve stability. Our hypothesis is that using a ratcheting technology in a fusion procedure will maximize compression forces across the fusion surface with axial loading. This study investigates the mechanical properties (axial compression, stress shielding and load to failure) of this ratcheting nail design relative to the current designs used in clinical practice (threaded and solid nails).

## Materials and methods

**Figure 2:**
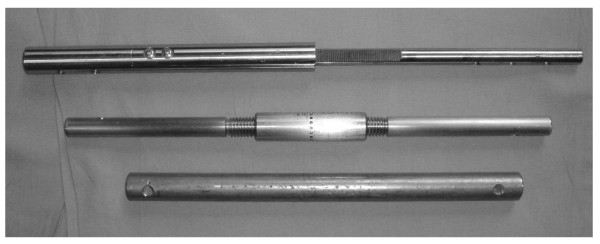
**Ratcheted nail, threaded nail and solid nail is shown in the photograph**. The ratcheting nail provides dynamic compression with axial loading while the threaded nail allows manual compression with each turn of the thread. The solid nail does not allow any type of compression across the fusion site.

To compare the properties of this ratcheting nail to the mechanical properties of existing designs for fusion nails, a total of three different IM nails were manufactured. Prototype #1 was a solid nail that is commonly used in clinical practice. Prototype #2 uses a threaded interlocking device similar to that used in currently marketed knee fusion nail designs to provide compression at the time of implantation. Prototype #3 is the novel IM nail with the ratcheting design (Figure 2).

**Figure 3:**
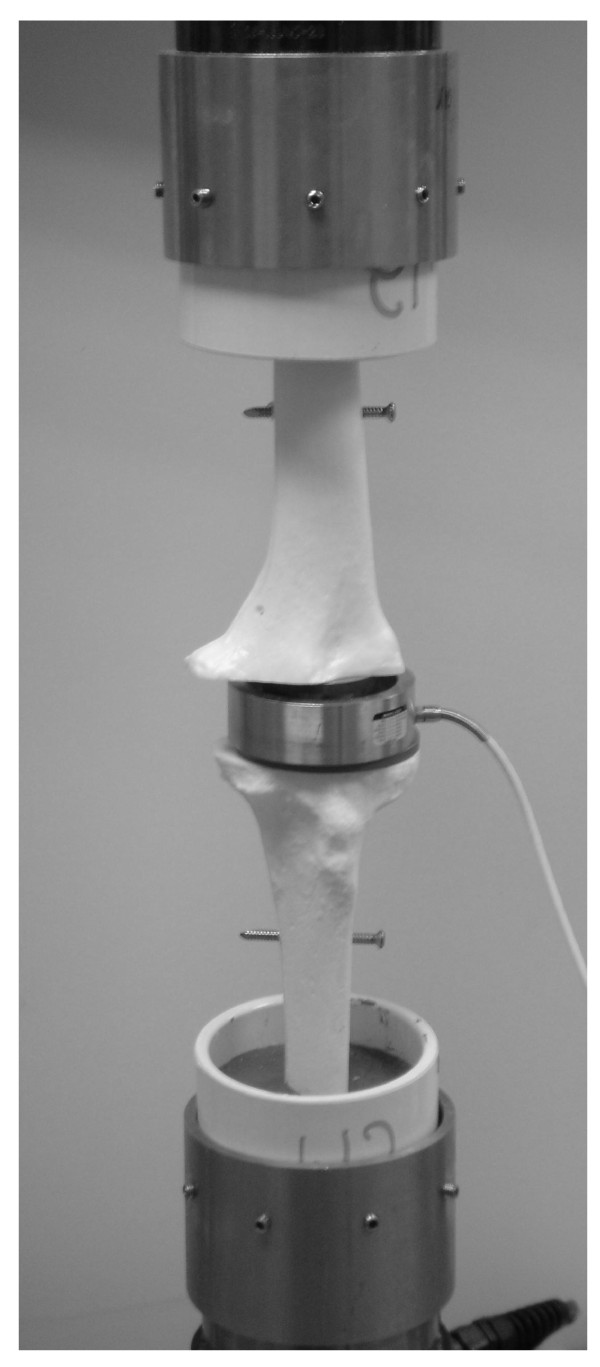
**Test construct loaded in MTS machine with the sawbone potted in cement with PVC pipes at both the proximal femur and distal tibia**. After insertion of the nail (solid, threaded or ratcheting) a load cell was placed flush to the fusion surface for the mechanical testing.

A knee design sawbone (foam cortical shell bone model, Pacific Research, Inc) fusion model was used to test our hypothesis (by utilizing a ratcheting design in a fusion nail compression forces across the fusion surface can be maximized with axial loading). The distal femur and the proximal tibia were cut in a manner consistent with standard knee fusion. A femoral and a tibial cutting jig were created to ensure uniformity of bone resection and that the surfaces were flush to each other. After preparation of the fusion surface, the sawbones were then potted into PVC (polyvinyl chloride) pipe caps using potting cement (Quick Crete Products, Inc. Norco, CA 92860) (Figure 3).

The three prototypes were inserted into the fusion construct in a manner replicating *in-vivo *surgical technique. The IM nail was first inserted retrograde into the femur and statically locked. Then the nail was inserted into the tibial canal in an antegrade direction. A washer-type load cell (Futek LTH 500) was used to separate the fusion surfaces. For the solid nail, manual compression was applied with a pointed tenaculum clamp (an instrument used in the operating room to assist in fracture reduction) prior to statically locking the distal aspect of the nail into the tibia with a screw. With the threaded nail, compression was also applied with a pointed tenaculum clamp before locking. Then, a hexagonal wrench was used to rotate the threaded locking mechanism to provide further compression. For the ratcheting nail, after locking the female and male components into the femur and tibia, respectively, the components were engaged and maximally ratcheted together by hand and with a pointed tenaculum clamp.

Mechanical testing was conducted using a Mini-Bionix 858 test frame (MTS, Inc. Eden Prairie, MN 55344). Load was measured at the site of compression using a washer-type load cell (Figure 3) with a central hole rated to 2000 lbs of failure (Futek, Inc. Irvine, CA). Tests were completed in displacement control mode (3 mm and 6 mm). Total compressive force, in-joint compressive force, distance, and time at a rate of 1 data point per 0.25 seconds were all recorded by computer read-out. The displacement position was held for ten seconds and the load was then removed from the system. Each prototype nail was tested in three sawbone knee constructs and data was collected for each test run. The resultant load across the fusion surface at the completion of each test cycle (as recorded by the load cell) was measured. This data point was then compared to the load reflected across the load cell after manual compression (initial load) to determine the percent of initial load across the fusion site. The average percent of initial load was then calculated for each of the three nail designs (Table 1) and standard deviation was also calculated.

**Table 1:** Data for percent initial load of each test construct.

	% initial load 3 mm	% initial load 6 mm
Solid 1	96	75.9

Solid 2	103.3	132.6

Solid 3	372	352

Solid Avg.	190.4	186.8

Solid S.D.	140.7	130.4

		

Threaded 1	72.8	50.6

Threaded 2	80.9	62.8

Threaded 3	88.3	75.5

Threaded Avg.	80.7	63.0

Threaded S.D.	6.9	11.1

		

Ratcheting 1	42.5	855

Ratcheting 2	99.4	517.1

Ratcheting 3	717.39	1117.4

Ratcheting Avg	286.4	829.8

Ratcheting S.D.	329.0	269.1

Stress shielding data were also calculated by recording the maximum load applied to the system by the test frame and comparing it to the load cell measurement of compression at that maximum external force. This value was recorded as percentage of the maximum load not reflected at the fusion surface (Table 2). A lower percentage thus reflects less stress shielding. All results were analyzed statistically using the Student t-test with significance set at p < 0.05.

**Table 2:** Data for stress shielding (SS) expressed as percent of initial load not reflected at fusion surface.

	SS 3 mm	SS 6 mm
Solid 1	33.0	32.0

Solid 2	41.0	28.6

Solid 3	30.4	25.4

Solid Avg.	34.8	28.7

Solid S.D.	4.9	3.0

		

Threaded 1	54.5	48.6

Threaded 2	29.1	33.7

Threaded 3	37.2	40.5

Threaded Avg.	40.3	40.9

Threaded S.D.	11.6	6.7

		

Ratcheting 1	29.0	11.5

Ratcheting 2	22.6	12.2

Ratcheting 3	3.8	10.7

Ratcheting Avg	18.5	11.5

Ratcheting S.D.	11.7	11.5

Load of failure were conducted with a mechanical test frame in axial compression (Admet Model 2611, Expert load frame, Norwood, MA) under load control using specimens gapped to a fixed distance. Load versus displacement curves were generated for each of the prototype nail. Nails were tested at 10 lbs/second to a maximum displacement of 1 cm. To account for the thread screw and ratcheting mechanism in prototype 2 and 3, we tightened the screw mechanism maximally and compressed the ratchet to its maximal point before application of load. For the purpose of this test, load of failure was defined as displacement of greater than 1 cm or an abrupt drop in the load displacement curve indicating the nails inability to transmit load.

## Results

The solid and threaded nails did not have large increases in initial compression load across the fusion surface after the 3 mm and 6 mm displacement trials. However, the ratcheting nail did have a significant increase in initial compression, especially at 6 mm of displacement. In the 3 mm displacement trials, we found no significant difference in maintenance of initial load for the solid vs. ratcheting nail (*p *= 0.70) or the threaded vs. ratcheting nail (*p *= 0.40). Data for the 6 mm displacement trials, however, showed a significant increase in the initial compressive load maintained across the fusion surface with the ratcheting nail versus the solid nail (829.8% vs. 186.8%, p = 0.03) and versus the threaded nail (829.8% vs. 63.0%, p = 0.01).

The stress shielding results of the solid and threaded nails were compared to the ratcheting nail. No statistically significant difference was found when comparing stress-shielding for the 3 mm displacement trials between the ratcheting vs. solid nail (*p *= 0.12) or the ratcheting vs. threaded nail (*p *= 0.11). For the 6 mm displacement trials, however, there was a significant decrease in stress-shielding through the system when the ratcheting nail was compared to the solid nail (11.5% vs. 28.7%, *p *= 0.001) and the threaded nail (11.5% vs. 40.9%, *p *= 0.002).

**Figure 4:**
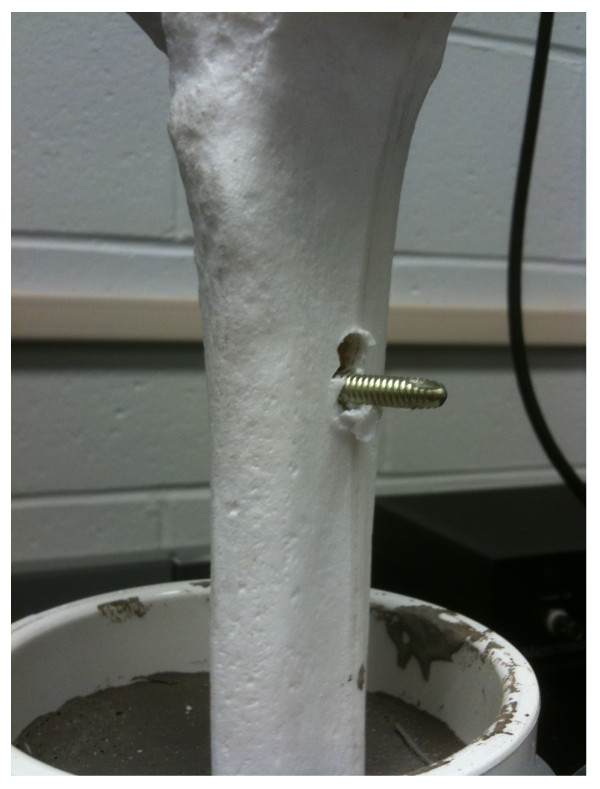
**Failure of the distal interlocking screw at the tibia observed with axial load**. This is the primary mode of failure in all tested constructs.

Load to failure in axial compression for the ratcheting nail was 599.0 lbs, threaded nail was 508.8 lbs, and solid nail at 688.1 lbs. In each case, the specimens failed at the interlocking screws (Figure 4).

## Discussion

The goal of joint arthrodesis is to create a painless and stable union between the intended fusion surfaces as a means to improve a patient's function and outcome [2,3,21]. When fusion is not achieved (non-union), pain and disability commonly persist. Knee arthrodesis has been performed since the 1900s to treat conditions associated with arthritis, sepsis, Charcot neuropathy, and reconstruction following tumor resection [3,21]. With the success of modern total knee arthroplasty (TKA), the current indication for knee arthrodesis have been narrowed to primarily include patients who have failed TKA with sepsis, significant bone loss, or instability in an unreconstructable knee [1,3,5-9,12,16,20,21]. The fusion rate following knee arthrodesis is significantly higher for patients with post traumatic or rheumatoid arthritis [22,23] (>95%) in comparison to patients with the diagnosis of charcot arthropathy or infection after TKA [2,3,6,7,9,16,24] (30% to 100%). Tibiotalar Calcaneal (TCC) fusion is a salvage procedure used to treat failed total ankle arthroplasty, sepsis, post traumatic arthritis, or hindfoot deformities [4,6,10,11]. Up to 50% complication rate have been reported in the literature with TCC fusion that include infection, nonunion, malunion, wound complications, and amputation [10]. Therefore it is essential to improve the current design of fusion nails to maximize the stability of the fusion surface to improve clinical healing.

This investigation was performed with the goal of improving the currently commercially available fusion nails by utilizing a novel ratcheting device that could be used to allow dynamic loading across an intended site of joint fusion. The data demonstrated a statistically significant improvement in initial load across the fusion surface with the ratcheting nail (Prototype #3) when compared to the solid and threaded nails in the 6-mm displacement load trials. As the teeth in the ratcheting device engaged, the amount of compression applied was maintained and would allow increased compression forces across the fusion site. When compression displacement of only three millimeters was applied, an advantage was not seen with the ratcheting device. This finding was primarily because the amount of compression was insufficient to advance the ratchet mechanism. However, analysis of the 3-mm displacement data points for the ratcheting nail (Table 1) demonstrates an aberrantly high value for one trial (Ratcheting #3). In this particular trial, the teeth of the ratchet mechanism were able to advance with only 3-mm of displacement. The teeth of this ratcheting nail can be engineered to be at variable length that would allow for controlled displacement with axial loading.

There is a distinct advantage in the ratcheting mechanism when compared to the currently clinically available nails. With sufficient axial load, the ratchet will advance. Therefore, it will always maintain a significant amount of compressive force at the fusion surface, even with subsidence or collapse of bone at the fusion surface over time. Dynamization or axial compression of transverse osteotomies has been shown to increase both the torsional stability and maximal torque of the fracture site when compared to locked rigid control in a canine model [25]. Both the solid and threaded nail design will not allow further advancement of the nail with axial loading as they are both statically locked devices. Furthermore, the stress shielding data for the 6-mm displacement trials demonstrated a significant (*p *< 0.05) decrease in stress shielding for the ratcheting nail as compared to both the solid and the threaded nails. This decrease in stress shielding is likely a result of the dynamic nature of the ratcheting design which allows for controlled axial compression at the fusion surface. The solid and the threaded nail designs, by comparison, were statically locked and thus provided a greater degree of stress shielding. This decrease in stress shielding may also be an advantage for improved bone healing and fusion [26,27].

To further investigate the mechanical properties of the ratcheting nail, we tested the three prototypes to failure in axial compression. We chose to test them in compression because this is the likely mode of primary loading. However, this may not represent true physiologic loads as the nails placed clinically would likely be subjected to both torsional and moment loads as well as pure axial loading. For the purpose of this test, load of failure was defined as displacement of greater than 1 cm or an abrupt drop in the load displacement curve indicating the nails inability to transmit load. In each case, the specimens failed at the interlocking screws. This is not surprising as in clinical situations; locking screw failure is the most commonly seen mode of failure after long bone nonunion or fracture [28]. However, each specimen was able to withstand axial loads of greater than 500 lbs prior to failure. While this test does not address potential weakness of the ratcheting nail after cyclic loading, it does confirm that the bone-implant interface is the weakest aspect of the construct as evidenced by failure of the locking screw.

The major limitation of this study is that this is an in vitro biomechanical analysis characterizing only the axial compression, stress shielding, and load to failure of this novel ratcheting fusion nail. Evaluating the axial compressive properties without testing torsion and bending is not sufficient to fully evaluate a fusion fixation nail. In the clinical setting, there are more forces involved at the fusion site and without further mechanical testing of this nail, clinical trials can not be performed. We believe that by increasing the compression forces across the fusion surface with axial loading while minimizing stress shielding will increase clinical rates of knee or TCC fusion, however, this statement along with characterizing the torsion and bending properties of this nail needs to be further investigated.

## Conclusion

This data, while preliminary, suggests that a ratcheting device may have useful clinical applications. A statistically significant increase in the load maintained across the fusion surface and decrease in the stress shielding of the fusion construct with a ratcheting nail was seen with 6 mm of displacement. The preliminary data from this study validates the concept that a ratchet mechanism may be a viable design option for a fusion nail to maximize compression and facilitate union. However, further experiments in the future will be performed in cadaver models to further characterize the mechanical properties (torsion and bending) of this ratcheting nail before clinical experimentations.

## Competing interests

The authors declare that they have no competing interests.

## Authors' contributions

XL, JM and JW have contributed to the data collection/interpretation, mechanical testing and drafting/revising of the manuscript. DW and KB have contributed to the mechanical testing and mechanical evaluation of the fusion nails. JW have contributed to the conception and design of this particular ratcheting arthrodesis nail. All authors approved the final manuscript.
